# Rapid Syphilis Testing Is Cost-Effective Even in Low-Prevalence Settings: The CISNE-PERU Experience

**DOI:** 10.1371/journal.pone.0149568

**Published:** 2016-03-07

**Authors:** Patricia Mallma, Patricia Garcia, Cesar Carcamo, Sergio Torres-Rueda, Rosanna Peeling, David Mabey, Fern Terris-Prestholt

**Affiliations:** 1 Epidemiology, STD/HIV Unit, School of Public Health, Universidad Peruana Cayetano Heredia, Lima, Peru; 2 London School of Hygiene and Tropical Medicine, London, United Kingdom; California Department of Public Health, UNITED STATES

## Abstract

Studies have addressed cost-effectiveness of syphilis testing of pregnant women in high-prevalence settings. This study compares costs of rapid syphilis testing (RST) with laboratory-based rapid plasma reagin (RPR) tests in low-prevalence settings in Peru. The RST was introduced in a tertiary-level maternity hospital and in the Ventanilla Network of primary health centers, where syphilis prevalence is approximately 1%. The costs per woman tested and treated with RST at the hospital were $2.70 and $369 respectively compared with $3.60 and $740 for RPR. For the Ventanilla Network the costs per woman tested and treated with RST were $3.19 and $295 respectively compared with $5.55 and $1454 for RPR. The cost per DALY averted using RST was $46 vs. $109 for RPR. RST showed lower costs compared to the WHO standard costs per DALY ($64). Findings suggest syphilis screening with RST is cost-effective in low-prevalence settings.

## Introduction

Syphilis remains an important global health issue. The World Health Organization (WHO) estimates that every year 1.5 million pregnant women are infected with syphilis [1]. At least half of untreated infected pregnant women will have serious adverse events associated with syphilis, including stillbirths, neonatal or early infant deaths, and infants with clinical signs of the disease[1]. Penicillin is the treatment of choice and can prevent complications and congenital syphilis if pregnant women are treated early in pregnancy, ideally before 20 weeks of gestation [2]. Although almost every country in the world has policies or guidelines related to syphilis screening in antenatal care (ANC) implementation is still a problem in many settings; women are often not screened, results are not always available and infected women fail to undergo treatment[3].

Rapid syphilis tests (RSTs) are now available for use at the point of care. Since results are available in 15–20 minutes, they allow for same-day testing and treatment. Most currently available RSTs are treponemal tests, which detect antibodies to treponemal antigens and have high sensitivity and specificity when compared to non-treponemal screening tests such as the rapid plasma regain (RPR) test in low prevalence populations such as pregnant women. RSTs therefore present an opportunity for improving screening programs in ANC, especially in resource limited settings [4–5].

Although there is some published literature on costs and cost-effectiveness of rapid syphilis testing, almost all of it relates to African countries. Limited information exists on settings with relatively low maternal syphilis prevalence, such as most Latin American countries. Furthermore, work on the subject tends to be centred on models that rely heavily on assumptions (e.g sensitivities and specificities, coverage of screening and treatment, etc) rather than on primary data [6–16].

As part of a six-country implementation study [17], rapid syphilis tests SD BIOLINE Syphilis 3.0^®^ (Standard Diagnostics, Korea) were introduced in Peru in 2010, through the CISNE Project and, soon after, their use became national policy [18]. The objective of this paper is to compare the cost-effectiveness analysis of a point-of-care test with the standard of care (rapid plasma reagin (RPR) test) for reproductive care services in Peru, a setting with low prevalence of maternal syphilis, and to compare their cost-effectiveness in three services: antenatal care (ANC), delivery and miscarriage services.

## Methods

RST was introduced in two low prevalence (≤1%) settings in Peru: (1) a tertiary-level referral maternity hospital with the largest number of deliveries in the country, the Instituto Nacional Materno Perinatal (INMP); and (2) the Ventanilla Network, consisting of 16 health facilities (which include 15 health centers and one small hospital), where RST was implemented together with rapid HIV testing: “two tests one stick, or two for one”[18]. The Peruvian National Guidelines recommended syphilis screening with RPR for all pregnant women at the first ANC visit and also for pregnant women seen at miscarriage services and delivery/emergency services, mainly to detect congenital syphilis cases and treat them appropriately (with Benzathine penicillin 1.2 million units IM every week for three weeks) and to prevent future new cases of congenital syphilis. For the cost-effectiveness analysis we included INMP and five of the health facilities of the Ventanilla Network. We classified all the health facilities within the Ventanilla Network into four types: (1) type I, small hospital with laboratory; (2) type II, health center with laboratory; (3) type III, health center without a laboratory (blood is drawn at the facility, sent daily to type II facilities where samples are processed and returned to type III facility), and (4) type IV, health center without a laboratory (patients are referred to type II facility to have blood drawn, they have to return to the type II facility at a later date to pick up results, and then go back to the type IV facility to see their provider).

For the study we randomly chose one facility each from type I (the only one of this type), type III, and type IV, and two facilities from type II. All the costing data were obtained in early 2010. Patient flows for RST and RPR were mapped. Cost data were collected for both RPR and RST based on the Guidelines for Cost Effectiveness Analysis of Syphilis Screening Strategies [19].

### Cost Analysis

Costs considered for RPR included: laboratory supplies, space rental, allocated staff salaries and treatment costs. These costs were associated with blood sampling, processing of the blood sample and reading of the RPR, and with treatment of a positive index case and of one partner. Costs considered for RST included: expenses for the implementation activities (advocacy meetings with authorities, training, supervision, and monitoring) and the cost of implementing the QA system. Costs of supplies, such as lancets, alcohol swabs, kits (cassette and buffer), gloves, biosecurity devices, supplies for treatment of the positive index case and partner (assuming all partners receive treatment), among others, as well as allocated staff salaries, were also included. For the Ventanilla Network facilities, costs of personnel time and building space were allocated equally between HIV and syphilis screening, as RST was rolled out alongside rapid HIV testing.

The information on unit price of materials and supplies, and costs associated with personnel, laboratory equipment, electricity and water consumption of the respective health facilities, were provided by offices of the Callao Regional Health Directorate for the Ventanilla Network. For INMP, data were obtained through the logistics office of the hospital. Each of the offices was visited, staff responsible for purchasing supplies were interviewed and information from invoices and reports was collected. Data on training costs associated with RST were collected through bills, invoices of supplies and reagents purchased by the CISNE Project provided by the project manager. Data on associated costs of training were obtained by allocating salaries of project staff involved in the activities.

### Project Outputs and Unit Costs

Project outputs included: number of pregnant women tested in each of the facilities, number of women with reactive tests for syphilis and number of women treated. The data for RST was obtained from records of the CISNE Project implementation, which took place from January to November 2010 (data was projected for the whole year). Data for RPR were obtained from 2009 statistical reports from each health facility. The economic unit cost per woman tested and the cost per woman treated were both calculated for each service within each facility. Based on the model presented by Terris-Prestholt et al [20] true cases treated were estimated. This model used quality assurance data from Ventanilla to assess the performance of the RPR and RST tests in this setting. Based on this model, the percentage of true cases treated among women who were tested and treated is 90.7% with RST and 33% with RPR. These figures were obtained by comparing field performances against laboratory-based RPR and Treponema Pallidum Hemagglutination Assay tests (TPHA). Disability-adjusted life years (DALY) averted were only calculated for adverse outcomes in infants, and estimated by the number of women seen in ANC, prevalences, diagnostic performance and treatment rates. Using standard DALY inputs (3% discount rate and stillbirth considered a full life lost), each true case treated is assumed to avert 5.73 DALYs in Peru. The estimation of DALYs for this study followed the methods presented by Terris-Prestholt et al [20].

### Sensitivity Analysis

A univariate sensitivity analysis was performed to determine the impact of uncertainty of costs and output. Factors included were: discount rate (variation from 3% to 9%); working hours of the health workers (variation from 6 to 8 hours per day); screening rates for syphilis (variation from 57% to 100%); exchange rate (US dollars (USD) to Peruvian new soles (PNS) with a variation of 2.638 to 2.910 PNS:USD); syphilis prevalence (variation from 0.6% to 2.2%); building costs (increments of 22.1%); and health personnel salaries (both for screening and for treatment, variation between 0% to 50%).

The Ethics Committee at Universidad Peruana Cayetano Heredia reviewed and approved the main study (Approval number 55202). The current study did not involve participation of human beings. We have verified and confirmed that we have no identifying information for patients.

## Results

For 2010 (January to December) we projected a total of 17,919 and 3,908 women screened with the RST at INMP and at the five health facilities from the Ventanilla Network, respectively. Women were seen at ANC services, miscarriage services, and delivery and emergency services. Screening coverage for RST was 95% at INMP and 90% at the five facilities of the Ventanilla Network. For RPR estimated coverage was lower: 62% at INMP and 34% for the Ventanilla Network. Syphilis prevalence with RST was 0.9% at INMP and 1.2% at the Ventanilla Network health facilities. The prevalences observed with RPR were lower: 0.6% at INMP and 0.5% at the Ventanilla Network.

Regarding treatment, 91% and 90% of RST-positive women from INMP and from the Ventanilla Network were treated, respectively. Eighty-three percent of RPR-positive women received appropriate treatment at both INMP and the Ventanilla Network (Table 1).

**Table 1:** Screening and treatment outputs.

RST Outputs (2010)	INMP	Facility A*	Facility B	Facility C	Facility D	Facility E	All five facilities
Total of women in ANC/MS/DE**	17919	2509	649	268	214	268	3908
Women Tested	17084	2287	547	232	206	241	3513
Women Positive	150	26	8	5	1	3	43
Women who required treatment***	137	26	7	5	1	3	42
Women Treated	125	23	7	4	1	3	38
% of women tested	95%	91%	84%	87%	96%	90%	90%
% Reactive	0.9%	1.1%	1.5%	2.2%	0.5%	1.2%	1.2%
% of positives treated	91%	88%	100%	80%	100%	100%	90%
Real cases treated in women****	113	21	6	4	1	3	35
**RPR Outputs (2010)**							
Total of women in ANC/MS/DE**	17919	2509	649	268	214	268	3908
Women Tested	11113	591	296	147	123	154	1311
Women Positive	65	3.8	0.8	0.4	0.4	0.5	6
Women Treated	54	2.9	0.7	0.4	0.3	0.4	5
% of women tested	62%	24%	46%	55%	57%	57%	34%
% Reactive	0.6%	0.6%	0.3%	0.3%	0.3%	0.3%	0.5%
% of positives treated	83%	76%	88%	100%	75%	80%	83%
Real cases treated in women****	18	1.0	0.2	0.1	0.1	0.1	1.5

* Establishments A is type I, B and C are type II, D is type III and E type IV. Facilities A through E are located at Ventanilla Network

**ANC: antenatal care services. MS: miscarriage services. DE: Delivery and emergency services.

*** Some women had a clear history of previous treatment (recent) and it was deemed they did not need treatment.

**** Calculated using model from Terris-Prestholt et al. (20)

### Costs

**Fig 1:**
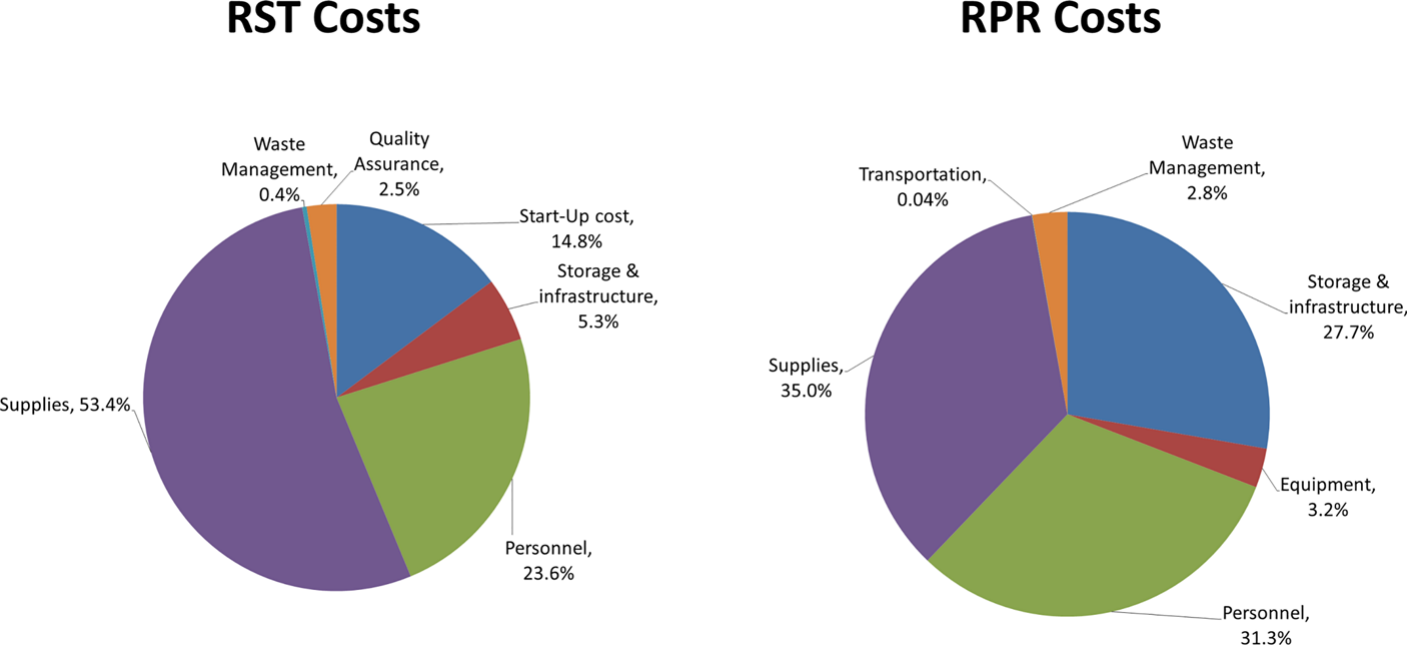
RST and RPR cost components.

Total economic costs associated with screening with RST and subsequent treatment ranged from $1,008 to $46,067 per facility. Supplies accounted for the greatest share of total costs (53.4%), followed by the personnel costs (23.6%) and start-up costs (14.8%). Quality assurance costs represented only 2.5% of costs. For RPR the total economic costs of screening and treatment ranged from $403 to $39,957. Supplies costs accounted for 35% of total costs, followed by personnel costs (31.3%) and store and building costs (27.7%) (Table 2) (Fig 1).

**Table 2:** Total screening and treatment costs of RST and RPR (USD).

**RST costs ($USD)**	**INMP**	Facility A*	**Facility B**	**Facility C**	**Facility D**	**Facility E**	**All five facilities**
**Start-up and training costs**	**$4,369**	**$1,580**	**$894**	**$610**	**$465**	**$539**	**$4,088**
**Testing and treatment costs**	**$41,261**	**$3,662**	**$969**	**$538**	**$473**	**$496**	**$6,138**
Capital	$2,493	$238	$74	$95	$95	$60	$562
Personnel	$12,323	$597	$212	$146	$125	$137	$1,217
Supplies	$26,257	$2,815	$679	$294	$250	$296	$4,334
Waste management	$188	$12	$4	$3	$3	$3	$25
**Quality Assurance costs**	**$437**	**$486**	**$190**	**$158**	**$70**	**$80**	**$984**
**TOTAL COSTS RST**	***$46*,*067***	***$5*,*728***	***$2*,*053***	***$1*,*306***	***$1*,*008***	***$1*,*115***	***$11*,*210***
**Unit Costs**							
Cost per woman tested RST**	**$2.70**	**$2.50**	**$3.75**	**$5.63**	**$4.89**	**$4.63**	**$3.19**
Cost per woman treated RST**	**$369**	**$249**	**$293**	**$327**	**$1008**	**$372**	**$295**
**Cost per true case treated** ***	**$308**	**$295**	**$390**	**$725**	**$508**	**$478**	**$366**
**RST costs ($USD)**	**INMP**	Facility A*	**Facility B**	**Facility C**	**Facility D**	**Facility E**	**All five facilities**
**Start-up and training costs**							
**Testing and treatment costs**	**$39957**	**$3643**	**$1522**	**$1067**	**$636**	**$403**	**$7271**
Capital	$11335	$1775	$645	$614	$162	$47	$3243
Personnel	$12243	$1199	$518	$295	$336	$185	$2533
Supplies	$15271	$593	$293	$125	$101	$153	$1265
Transportation					$18		$18
Waste management	$1108	$76	$66	$33	$19	$18	$212
**Quality Assurance costs**							
**TOTAL COSTS RPR**	***$39957***	***$3643***	***$1522***	***$1067***	***$636***	***$403***	***$7271***
**Unit Costs**							
**Cost per woman tested RPR**	**$3.60**	**$6.16**	**$5.14**	**$7.26**	**$5.17**	**$2.62**	**$5.55**
**Cost per woman treated RPR**	**$740**	**$1,256**	**$2,174**	**$2,668**	**$2,120**	**$1,008**	**$1,454**
Cost per true case treated***	**$621**	**$1142**	**$831**	**$1038**	**$997**	**$473**	**$946**

*Facilities A through E are located at Ventanilla Network

**Includes start-up and training costs

***Calculated using model from Terris-Prestholt et al. (20)

### Unit Costs

The cost per woman tested at INMP was estimated to be $2.70 for RST and $3.60 for RPR. The cost per woman treated was $369 for RST and $740 for RPR.

**Fig 2:**
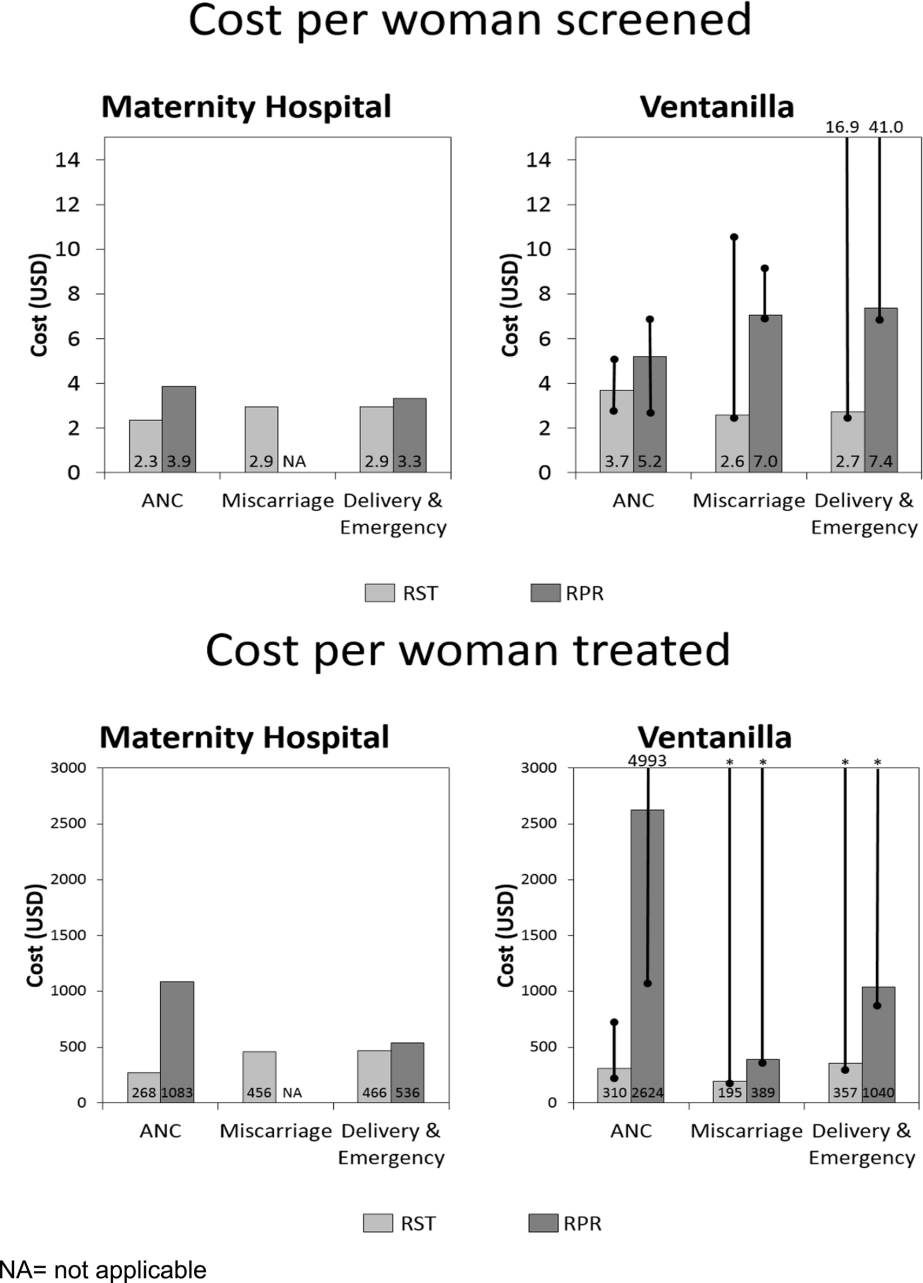
Costs per woman screened and treated, by service comparing RST vs RPR at INMP and Ventanilla Network.

At the Ventanilla Network, the cost per woman tested (average of the five establishments) was $3.19 for RST and $5.55 for RPR. The cost per woman treated was $295 for RST and $1,454 for RPR. Costs per woman tested and treated by type of service (ANC, miscarriage services and delivery/emergency) for INMP and for the Ventanilla Network for both RST and RPR are compared in Fig 2.

The cost per DALY averted by screening in ANC was $46 for RST and $109 for RPR. These number do not change much when reducing the proportion of stillbirths from 4.6% to 0% ($42 and $100 for RST and RPR respectively). Although either intervention could judged as very cost-effective compared with the Peruvian GDP per capita for 2011 of $5759 USD, screening with RST was more cost-effective than with RPR at all health facilities.

**Fig 3:**
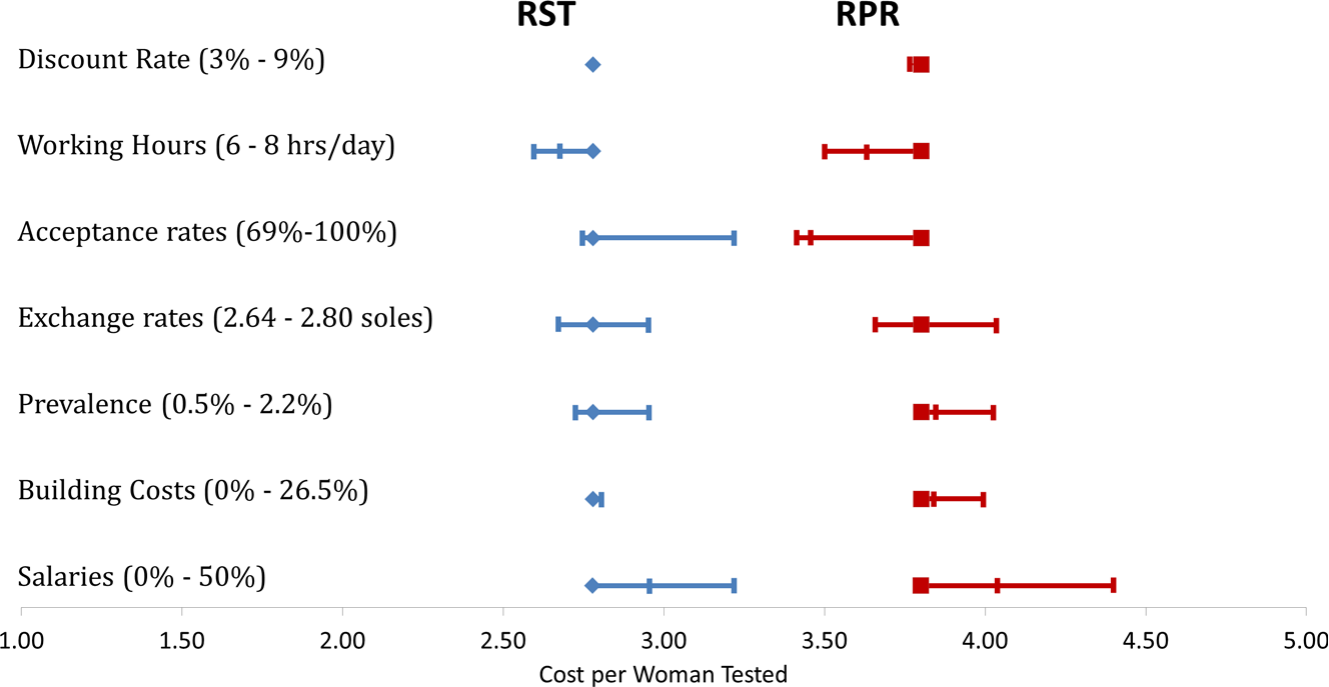
Univariate sensitivity analysis.

Cost per woman tested was used in the univariate sensitivity analysis. For RST, the cost per woman screened increased when the number of positive cases increased and when salaries increased. However, when the coverage of screening increased the cost per woman tested decreased. For RPR costs decreased when the number of hours worked and the screening coverage increased. But if the salaries or the prevalence of syphilis increased, the cost per women tested also increased (Table 3, Fig 3). Cost-effectiveness of RST is thus sensitive to the coverage of screening, disease prevalence and salaries, while RPR is also sensitive to the number of working hours.

**Table 3:** Univariate sensitivity analysis.

Parameters varied	Cost per woman tested (USD)		Parameters varied	Cost per woman tested (USD)	
	RST	RPR		RST	RPR
**Discount Rate**			**Prevalence**		
3%	2.78	3.78	0.6%	2.73	3.80
6%	2.78	3.79	0.9%	2.78	3.85
9%	2.78	3.80	2.2%	2.95	4.03
**Working Hours**			**Building Costs**		
6 hours	2.78	3.80	100.0%	2.78	3.80
7 hours	2.68	3.63	104.4%	2.79	3.84
8 hours	2.60	3.50	122.1%	2.81	3.99
**Acceptance rates**			**Salaries**		
57%	3.22	3.80	100%	2.78	3.80
94%	2.78	3.46	120%	2.96	4.04
100%	2.75	3.42	150%	3.22	4.40
**Exchange rates**					
1USD = 2.800 soles	2.78	3.80			
1USD = 2.910 soles	2.68	3.66			
1USD = 2.638 soles	2.95	4.03			

## Discussion

Peruvian reproductive health guidelines recommend syphilis screening for all women in ANC and for women attending reproductive health services: miscarriage services and delivery-emergency services. After the successful implementation of the rapid syphilis test in Peru [18], the guidelines promote using them to improve coverage of screening and treatment. Syphilis prevalence was found to be lower when using the RPR test (0.6% and 0.5% at the INMP and Ventanilla Network, respectively) compared to when the RST was used (0.9% and 1.2%, respectively). This difference was found in part to be related to the poor performance of the RPR. A QA evaluation of the RPR performance showed that half of the positive syphilis tests were missed by the RPR performed at the facilities due to several factors: lack of equipment (centrifuges and/or rotators), staff not following procedures adequately, and the poor quality of reagent and cards. Another reason to explain why RST detected more cases than RPR is that RST detects women with syphilis regardless of whether they have current infection or past treated infection. Prevalence of past syphilis infections is expected to be low, as women in ANC are relatively young and from a low risk population, thus they would not contribute much to overtreatment. Nevertheless, the benefit of improving treatment coverage among infected women outweighs the potential risk and costs of unnecessary treatment for some uninfected women. Since we have no good serological test to detect re-infection and the serious consequence of missing any reinfections, all women with a RST positive result should be treated. The screening and treatment coverage was also higher when using RST.

The economic comparisons did not include implementation, training or QA activities for RPR, since those activities had taken place long ago or were not done on a regular basis. Those costs were only included for RST in order to get a more accurate view of the start-up costs involved in introduction. Start-up costs for RST represented almost 15% of the economic costs but, compared with RPR, personnel costs were much lower in proportion (23.6% for RST vs 31.3% for RPR). Supplies made up the greatest share of RST costs (53.4%). It is important to highlight that the cost of RST for the country was significantly reduced by the introduction of a national-level procedure to purchase centrally (as one large national bulk purchase of RSTs) and using UNICEF’s procurement system, which led to much lower costs than purchasing them through national commercial distributors. Peru has adopted this system and Peruvian public health professionals are attempting to share lessons learned with other Latin American countries.

Cost per woman tested, cost per woman treated and cost per DALY averted with RST were lower compared with the costs related to RPR ($3.19, $295, and $46 for RST, and $5.55, $1454, and $109 for RPR, respectively). This suggests that even for a setting with low syphilis prevalence the RST is more cost-effective than the RPR.

Previous studies, mainly focusing on African countries and largely based on cost-modeling, have reported that cost per woman screened and/or cost per woman treated varied according to the costs included in the analysis or the prevalence of the disease [6,9–14]. For example, a study in Bolivia, which did not include implementation or QA costs, found that the average cost per woman tested was $1.48 and $1.91 using the RPR and a rapid strip test, respectively [7]. A study from Tanzania [15] showed an average cost per woman screened of $1.92 for RST and $2.32 for the RPR. The costs per woman treated were $21.35 for RST and $12.96 for the RPR. A total of 6362 women were tested with RST and only 224 with RPR, with reactivity rates ranging from 9% to 59%, depending of the clinic/site. The study concluded that RST was less expensive than RPR in the Tanzania setting. In most of the previous studies the costs per woman screened with RST was higher in comparison with the RPR, although the use of RST was deemed more cost-effective than that of the RPR because a higher proportion of women testing positive were treated when using a point of care test. Only the Tanzanian study found that the screening costs, in this high prevalence setting, were lower with RST than with RPR. Our study produced similar findings but in a low- prevalence setting (around 1%). These findings may resonate with other Latin American countries, most of which have similarly low prevalence rates.

A recent article modeled the cost, health impact, and cost-effectiveness of expanded syphilis screening and treatment in ANC for eight generic country case scenarios. Using syphilis prevalence as one of the factors, the study concluded that countries with high prevalence, low current service coverage, and high healthcare costs would benefit most from expanded screening and services. However, they also acknowledge the need of local epidemiologic and programmatic data to adjust the models [10]. The findings from our study show that there could even be important benefits in low-prevalence settings if screening coverage is improved by using RST.

The implementation of a QA system for screening with RST in our study represented a very small percent (2.5%) of the total economic costs. The QA costs included training on QA issues, preparation of the dried tube specimens [19], delivery of the packages to the clinics and the visits for external and internal QA and evaluations. On average the cost of the QA system per woman screened was $ 0.40 with a range from $ 0.03 to $ 0.68 (lower at the larger health establishments reflecting economies of scale). These costs were lower than those presented in the Tanzania study [15]. This could be explained by the differences in distances between remote establishments in Tanzania as opposed to the Ventanilla Network in Peru, and the frequency of the evaluations. Evaluations were reported monthly in Tanzania. In Peru, visits and monitoring were included in the implementation phase (which are reflected in start-up costs, not in the QA costs), the first external QA (evaluation of the performance of the health professionals reading the RST) was performed at the end of the training, and then repeated every six months. The internal evaluation (to assess the quality of the kits) was also performed every six months. It is clear that the costs and effects of a QA system will vary according to the intensity of the program. One important issue is to find the right balance between the need for QA, the budget available, the prevalence of the disease (the lower the prevalence, the higher the need of QA), staff turnover and the potential increased workload for the health system staff. QA external evaluations were carried out every six months as the initial QA showed very good performance, due to staff turnover occurring around every six months and to budget constraints. Although there were some initial problems attributed to presbyopia (farsightedness), these were resolved by recommending reading glasses to the health workers carrying out the tests. Further studies are needed to evaluate the ideal frequency of QA visits, which will need to be tailored to different settings.

There are some limitations to this study. Data on prevalence, screening, and treatment in relation to RPR were collected from different statistical reports at each health facility, which showed some inconsistencies. On the other hand RST data were collected prospectively. Costing data varied greatly by health center. Although different types of health centers were included, costs at other health facilities may vary and we cannot be sure how much. For the cost-effectiveness analysis we lumped together data from ANC, miscarriage and delivery/emergency services. We did not analyze how cost effective it is to screen these different populations, which are included in the National Peruvian Guidelines. That analysis was beyond the scope of this paper, since it will need the development of a model that incorporates other reproductive issues, however this is an interesting area of future work.

This study also has important strengths. It includes costs of implementation and QA, which are essential for countries interested in introducing RST. It also offers data for countries with low prevalences of syphilis, like most Latin American countries, showing that the implementation of RST is cost-effective, a crucial finding given that the region has taken on the challenge of eliminating congenital syphilis [21]. This study shows that RSTs could potentially be a key element in achieving this goal.

## Conclusions

Even in low prevalence settings (1% prevalence of maternal syphilis), syphilis screening using rapid syphilis testing is cost-effective and cheaper than RPR. Costs may vary depending on the size of the health facility, mainly due to economies of scale. The cost associated with implementing a QA system represented a small percent of the economic costs but this is critical for a well-functioning program.

## Supporting Information

S1 Table Assumptions for cost calculations. (DOCX)

S2 Table Cost inputs for RPR and RST. (DOCX)
