# Supplementary material for: Rapid Syphilis Testing Is Cost-Effective Even in Low-Prevalence Settings: The CISNE-PERU Experience
Source: PLoS One. 2016 Mar 7;11(3):e0149568. doi: 10.1371/journal.pone.0149568 (PMC4780822; doi:10.1371/journal.pone.0149568)
Supplement: S1 Table — (DOCX) [file pone.0149568.s001.docx]

**Supplementary appendix: Assumptions**

1. **For RST**

| Trained professional | At INMP 17 health professionals were trained. |
| --- | --- |
|  | At Ventanilla Network (within 5 health facilities that were included in the cost-effectiveness study 43) midwives were trained. |
| Meetings | Costs of meetings were made based on the number of services for each health facility. |
| Materials | According the number of RST performed at each service. |
| Monitoring/supervision | According the number of RST performed at each service. |
| QA | According the number of quality controls performed during the implementation of RST. |
| Incoming inspection | According of number of services at each health facility. |

1. **For infrastructure for RST and RPR**

| **Health facilities** | **Area (m^2^)** | **RST** | | **RPR** | |
| --- | --- | --- | --- | --- | --- |
|  |  | **% allocation** | **Cost  ($ per m^2^)** | **% allocation** | **Cost  ($ per m^2^)** |
| **INMP** | 37500 |  |  |  |  |
| Blood sample collection | 14.38 |  |  | 21% | 777 |
| Immunology laboratory | 27.3 |  |  | 15% | 777 |
| Central storage | 200 | According to screening | 777 | According to screening | 777 |
| Lab storage | 21 | According to screening | 777 | According to screening | 777 |
| **A** | 5139.35 |  |  |  |  |
| Blood sample collection | 7.35 |  |  | 30% | 100 |
| Microbiology | 16.25 |  |  | 15% | 100 |
| Lab storage | 12 | According to screening | 100 | According to screening | 100 |
| **B** | 911.34 |  |  |  |  |
| Blood sample collection | 2 |  |  | 30% | 50 |
| Laboratory | 22.14 |  |  | 30% | 50 |
| Lab storage (inside the lab) | 22.14 | According to screening | 50 | According to screening | 50 |
| **C** | 470.41 |  |  |  |  |
| Blood sample collection | 1 |  |  | 30% | 80 |
| Laboratory | 10.5 |  |  | 30% | 80 |
| Lab storage | 16 | According to screening | 80 | According to screening | 80 |
| **D** | 216.98 |  |  |  |  |
| Blood sample collection | 6.35 |  |  | 30% | 80 |
| OB/GYN examining room | 1 | According to screening | 80 |  |  |
| **E** | 245.95 |  |  |  |  |
| OB/GYN examining room | 1 | According to screening | 50 | NA | NA |
| *NA = not applicable* |  |  |  |  |  |
| ** Health facility E didn't collect sample for RPR test* | |  |  |  |  |

1. **For cost of human resources**

For both, RST and RPR, times were calculated for each stage of the process. For example in RPR, it took the medical technologist approximately 18 minutes to centrifuge, load samples in the card, rotate and interpret test results.
