# Supplementary material for: Rapid Syphilis Testing Is Cost-Effective Even in Low-Prevalence Settings: The CISNE-PERU Experience
Source: PLoS One. 2016 Mar 7;11(3):e0149568. doi: 10.1371/journal.pone.0149568 (PMC4780822; doi:10.1371/journal.pone.0149568)
Supplement: S2 Table — (DOCX) [file pone.0149568.s002.docx]

**Supplementary appendix: Cost inputs for RPR and RST**

Costs associated with RPR and RST are given in the following tables:

- For RPR
  - Blood sample collection
    - Supplies

| **Supplies for blood sample collection** | **Unit cost (USD) 2010** | |
| --- | --- | --- |
|  | **INMP** | **Ventanilla Network** |
| Vacutainer tubes | 0.35 | 0.20 |
| Disposable needle for vacutainer | 0.12 | 0.04 |
| Holder (bag of 100) | 14.29 | N/A |
| Tourniquets | 0.57 | 0.57 |
| Cotton (for one needle stick) (500 gr) | 4.21 | 4.21 |
| Alcohol (1 liter) | 1.92 | 1.92 |
| Surgical tape | 24.71 | 24.71 |
| Disposable gloves | 0.32 | 0.36 |
| Marker | 0.57 | 0.93 |
| **Supplies for waste management** |  |  |
| Biohazard containers | 12.50 | 12.50 |
| Biohazard bags | 0.18 | 0.18 |
| Biohazard bag holder | 4.64 | 4.64 |

- - - Personnel

| **Personnel involved in blood sample collection for RPR** | **Cost for month (USD) 2010** | |
| --- | --- | --- |
|  | **INMP** | **Ventanilla Network** |
| Lab tech | 357.14 |  |
| Medical tech in site A |  | 490.72 |
| Lab tech in site A |  | 196.43 |
| Medical tech in site B |  | 616.07 |
| Lab tech in site C |  | 643.67 |
| Nurse tech in site D |  | 551.09 |
| A, B, C, D are health facilities at Ventanilla Network |  |  |
|  |  |  |

- - - Facilities

| **Area for blood sample collection** | **Cost (USD) 2010** | |
| --- | --- | --- |
|  | **INMP** | **Ventanilla Network** |
| Site C |  | 24.00 |
| Site D |  | 152.40 |
| In the other facilities there are no special areas for blood sample collection | | |

- - - Transport (only for site D)

| **Material & personnel for transport** | **Unit cost (USD) 2010** | |
| --- | --- | --- |
|  | **INMP** | **Ventanilla Network** |
| Cooler |  | 28.57 |
| Transport (in public transportation) |  | 1.43 |
| Nurse tech (in charge of transporting blood samples for RPR) |  | 551.09 |

- - Blood sample processing
    - Supplies

| **Supplies for blood sample processing** | **Unit cost (USD) 2010** | |
| --- | --- | --- |
|  | **INMP** | **Ventanilla Network** |
| Tips | 0.11 | 0.13 |
| Marker | 0.57 | 0.93 |
| Disposable gloves | 0.32 | 0.36 |
| **Waste Management** |  |  |
| Biohazard bags | 0.18 | 0.18 |
| Biohazard containers | 12.50 | 12.50 |
| Biohazard bag holder | 4.64 | 4.64 |
| **Reagents** |  |  |
| RPR | 0.11 | 0.10 |
| TPHA | 2.86 | N/A |

- - - Laboratory equipment

| **Equipment** | **Unit cost (USD) 2010** | |
| --- | --- | --- |
|  | **INMP** | **Ventanilla Network** |
| Centrifuge | 5,248.57 | 2,068.93 |
| Micropipete | 1,767.86 | 321.43 |
| Rotator | 3,186.07 | 1,336.78 |
| Timer | 35.71 | N/A |
| Refrigerator | 821.07 | 642.86 |

- - - Personnel

| **Personnel involved in blood sample processing for RPR** | **Cost for month (USD) 2010** | |
| --- | --- | --- |
|  | **INMP** | **Ventanilla Network** |
| Medical Tech | 816.59 |  |
| Medical Tech in site A |  | 637.87 |
| Medical Tech in site B |  | 616.07 |
| Laboratory Tech in site C |  | 643.67 |

- - - Infrastructure

| **Area for blood sample processing** | **Cost (USD) 2010** | |  |
| --- | --- | --- | --- |
|  | **INMP** | **Ventanilla Network** |  |
| Area | 3181.82 |  |  |
| A facility |  | 360.00 |  |
| B facility |  | 332.10 |  |
| C facility |  | 252.00 |  |
| In the other facilities there are no special areas for blood sample processing | | |  |

- - - Others

| **Others** | **Monthly cost (USD) 2010** | |
| --- | --- | --- |
|  | **INMP** | **Ventanilla Network*** |
| Water | 23.75 |  |
| Electricity | 47.51 |  |
| Communications | 7.92 |  |
| Generator | 5.32 |  |
| Others (garbage disposal) | 6.79 |  |
| Water in site A |  | 1,014.93 |
| Electricity in site A |  | 2,787.25 |
| Electricity in site B |  | 593.04 |
| Water in site C |  | 1,098.25 |
| Electricity in site C |  | 227.14 |
| * Cost is all center |  |  |

- - Storage
    - Infraestructure

| **Area** | **Cost (USD) 2010** | |
| --- | --- | --- |
|  | **INMP** | **Ventanilla Network** |
| At central storage | 3108.00 |  |
| At the lab | 815.85 |  |
| At the lab in site A |  | 60.00 |
| At the lab in site B |  | 55.35 |
| At the lab in site C |  | 12.00 |

- - - Personnel

| **Personnel** | **Cost for month (USD) 2010** | |
| --- | --- | --- |
|  | **INMP** | **Ventanilla Network** |
| Administrative staff | 571.43 |  |
| Medical tech |  | 637.86 |
| Lab tech |  | 643.67 |

- - - Others

| **Others** | **Cost for month (USD) 2010** | |  |
| --- | --- | --- | --- |
|  | **INMP** | **Ventanilla Network*** |  |
| Water | 4.97 | N/A |  |
| Electricity | 283.46 | N/A |  |
| Communications | 16.98 | N/A |  |
| * In Ventanilla Network could not find this information | | |  |

- - Treatment
    - Drugs and supplies

| **Drugs and supplies** | **Unit cost (USD) 2010** | |
| --- | --- | --- |
|  | **INMP** | **Ventanilla Network** |
| Benzathine penicillin 2'400,000 UI | 0.59 | 0.43 |
| Syringes | 0.07 | 0.07 |
| Disposable needle (2 x patient) | 0.02 | 0.02 |
| Distilled water | 0.04 | 0.04 |
| Cotton (500gr) | 4.21 | 4.21 |
| Alcohol (1 liter) | 1.92 | 1.92 |

- - - Personnel

| **Personnel** | **Cost for month (USD) 2010** | |
| --- | --- | --- |
|  | **INMP** | **Ventanilla Network** |
| Physician | 1357.14 | 1166.24 |
| Nurse or midwife | 892.86 |  |
| Midwife |  | 500.83 |
| Nurse |  | 357.14 |
| Nurse tech |  | 381.05 |

- For RST
  - Capillary blood collection
    - Supplies and reagents

| **Supplies for capillary blood collection** | **Unit cost (USD) 2010** | |
| --- | --- | --- |
|  | **INMP** | **Ventanilla Network** |
| Lancet | 0.46 | 0.46 |
| Cotton (500 gr) | 4.21 | 4.21 |
| Alcohol swab (x100 uni.) | 3.43 | 3.43 |
| Disposable gloves (x100 uni.) | 5.54 | 5.54 |
| Marker | 0.57 | 0.57 |
| **Waste management** |  |  |
| Biohazard containers | 2.50 | 2.50 |
| Biohazard bags | 0.21 | 0.21 |
| Biohazard bag holder | 4.64 | 4.64 |
| **Reagents** |  |  |
| Cassettes (including capillary) with customs clearance | 0.85 | 0.85 |

- - - Laboratory equipment

| **Equipment** | **Unit cost (USD) 2010** | |
| --- | --- | --- |
|  | **INMP** | **Ventanilla Network** |
| Timer | 4.29 | 4.29 |

- - - Personnel

| **Personnel involved in capillary blood for rapid test syphilis** | **Cost for month (USD) 2010** | |
| --- | --- | --- |
|  | **INMP** | **Ventanilla Network** |
| Lab tech | 357.14 |  |
| Medical Tech | 816.59 |  |
| Midwife |  | 501.69 |

- - Treatment
    - Drugs and supplies

| **Drugs and supplies** | **Unit cost (USD) 2010** | |
| --- | --- | --- |
|  | **INMP** | **Ventanilla Network** |
| Benzathine penicillin 2'400,000 UI | 0.59 | 0.43 |
| Syringes | 0.07 | 0.07 |
| Disposable needle (2 x paciente) | 0.02 | 0.02 |
| Distilled water | 0.04 | 0.04 |
| Cotton (500gr) | 4.21 | 4.21 |
| Alcohol (1 liter) | 1.92 | 1.92 |

- - - Personnel

| **Personnel** | **Cost for month (USD) 2010** | |
| --- | --- | --- |
|  | **INMP** | **Ventanilla Network** |
| Physician | 1357.14 |  |
| Nurse or midwife | 892.86 |  |
| Physician or Midwife |  | 892.86 |
| Nurse tech |  | 381.05 |

- - Storage
    - Infrastructure

| **Storage** | **Cost (USD) 2010** | |
| --- | --- | --- |
|  | **INMP** | **Ventanilla Network** |
| Area used in INMP | 1631.00 |  |
| Area used in site A |  | 105.00 |
| Area used in emergency site A |  | 135.00 |
| Area used in site B |  | 75.00 |
| Area used in site C |  | 96.00 |
| Area used in site D |  | 96.00 |
| Area used in site E |  | 60.00 |

- - - Personnel

| **Personnel** | **Cost for month (USD) 2010** | |
| --- | --- | --- |
|  | **INMP** | **Ventanilla Network** |
| Lab staff | 571.43 |  |
| Midwife |  | 501.69 |

- - - Others

| **Others** | **Cost for month (USD) 2010** | |  |
| --- | --- | --- | --- |
|  | **INMP** | **Ventanilla Network*** |  |
| Light | 69.43 | N/A |  |
| * In Ventanilla Network could not find this information | | |  |

- - Training
    - Supplies and reagents

| **Materials and supplies** | **Unit cost (USD) 2010** | |
| --- | --- | --- |
|  | **INMP** | **Ventanilla Network** |
| Lancet | 0.46 | 0.46 |
| Alcohol swab (x100uni.) | 3.43 | 3.43 |
| Disposable gloves (x100uni.) | 5.54 | 5.54 |
| Biohazard bags | 0.21 | 0.21 |
| **Reagents** |  |  |
| Cassettes (includes capillary) | 0.85 | 0.85 |

- - - Personnel

| **Personnel** | **Cost for month (USD) 2010** | |
| --- | --- | --- |
|  | **INMP** | **Ventanilla Network** |
| Trainer | 1009.33 | 1009.33 |
| Trainee | 821.43 | 501.69 |

- - - Transport

| **Transport** | **Unit cost (USD) 2010** | |
| --- | --- | --- |
|  | **INMP** | **Ventanilla Network** |
| Transportation of trainer, materials and supplies | 10.71 | 10.71 |

- - Advocacy meetings with authorities
    - Personnel

| **Personnel** | **Cost for month (USD) 2010** | |
| --- | --- | --- |
|  | **INMP** | **Ventanilla Network** |
| Health professional | 1250.00 | 1250.00 |

- - Materials for advocacy
    - Materials

| **Materials** | **Unit cost (USD) 2010** | |
| --- | --- | --- |
|  | **INMP** | **Ventanilla Network** |
| Posters | 0.27 | 0.27 |
| Brochure for pregnant women | 0.14 | 0.14 |
| Brochure for health professional | 0.28 | 0.28 |
| Video | 137.50 | 137.50 |
| **Materials for implementation** |  |  |
| Counseling flip chart | 1.33 | 1.33 |
| Instructive screening | 0.54 | 0.54 |

- - Monitoring/supervision
    - Personnel

| **Personnel** | **Cost for month (USD) 2010** | |
| --- | --- | --- |
|  | **INMP** | **Ventanilla Network** |
| Midwife | 428.57 | 428.57 |

- - Incoming inspection
    - Supplies

| **Supplies** | **Unit cost (USD) 2010** | |
| --- | --- | --- |
|  | **INMP** | **Ventanilla Network** |
| Positive cryovial 1:2 | 1.12 | 1.12 |
| Negative cryovial | 1.00 | 1.00 |
| Cryovial with 700 uL buffer | 0.23 | 0.23 |
| Cassettes PRS | 0.85 | 0.85 |
| Disposables gloves | 0.11 | 0.11 |
| Transfer pipette | 0.10 | 0.10 |

- - - Personnel

| **Personnel** | **Cost for month (USD) 2010** | |
| --- | --- | --- |
|  | **INMP** | **Ventanilla Network** |
| Biologist | 714.29 | 714.29 |

- - Quality assurance
    - DTS preparation
      - Preparation of trypan blue

| **Trypan blue - stock 0.1% (15mL)** | **Cost per unit (USD) 2010** |
| --- | --- |
| **1. Buffer preparation PT (1 liter)** |  |
| Deionized water (1 Liter) | 2.86 |
| Phosphate buffered saline (1 sachet 11.8gr) | 8.15 |
| Disposable syringe (10ml) | 0.13 |
| Autoclavable bottles storage (500 mL) | 2.14 |
| Membrane filter | 1.43 |
| Autoclavable bottles storage (1 Liter) | 8.20 |
| Disposables gloves | 0.11 |
| **2. Preparation of trypan blue** |  |
| Trypan blue dye | 97.00 |
| PT buffer | 23.02 |
| Disposable tube (15 mL) | 0.50 |
| Aluminum paper | 2.86 |
| Disposable serological pipette (10 mL) | 0.39 |
| Disposables gloves | 0.11 |
| Disposable mask | 1.07 |

- DTS (+)

| **1. DTS preparation pool of 3 sera (4 vials)** | **Cost per unit (USD) 2010** |
| --- | --- |
| RPR determinations | 0.46 |
| TPPA determinations | 1.07 |
| Cryovials 2.0 mL. | 0.21 |
| Cryobox | 0.54 |
| Disposable tips 0.5-10 uL | 0.11 |
| Disposables tips 20- 200uL | 0.11 |
| Disposables tips 100- 1000uL | 0.11 |
| Disposables gloves (x100uni.) | 5.54 |
| Trypan blue - stock 0.1% (15mL) | 2.46 |
| Biohazard bags | 0.21 |
| **2. Evaluation of DTS (1 DTS)** |  |
| Phosphate buffered saline (1 liter) | 23.02 |
| Transfer pipette | 0.10 |
| Disposables gloves (x100uni.) | 5.54 |
| Cassette of RST | 0.85 |

- DTS (-)

| **1. DTS preparation for negative samples** | **Cost per unit (USD) 2010** |
| --- | --- |
| RPR determinations | 0.46 |
| TPPA determinations | 1.07 |
| Cryovials 2.0 mL. | 0.21 |
| Cryobox | 0.54 |
| Disposable tips 0.5-10 uL | 0.11 |
| Disposables tips 20- 200uL | 0.11 |
| Disposables tips 100- 1000uL | 0.11 |
| Disposables gloves (x100uni.) | 5.54 |
| Trypan blue - stock 0.1% (15mL) | 2.46 |
| Biohazard bags | 0.21 |
| **2. Evaluation of DTS (1 DTS)** |  |
| Phosphate buffered saline (1 liter) | 23.02 |
| Transfer pipette | 0.10 |
| Disposables gloves (x100uni.) | 5.54 |
| Cassette of RST | 0.85 |

- Laboratory equipment

| **DTS preparation equipment** | **Cost per unit (USD) 2010** |
| --- | --- |
| Rotator | 461.43 |
| Timer | 4.29 |
| Analytical scale | 5000.00 |
| pH meter | 2800.00 |
| Refrigerator | 428.57 |
| Freezer -20°C | 650.00 |
| Micropipette 0.5-10uL | 283.19 |
| Micropipette 20-200uL | 283.19 |
| Micropipette 100-1000uL | 283.19 |
| Printer | 232.14 |

- Infrastructure

| **Building Space** | **Cost (USD) 2010** |
| --- | --- |
| Lab for sample preparation | 68.49 |

- Others

| **Others** | **Cost (USD) 2010** |
| --- | --- |
| Water | 17.6 |
| Electricity |  |

- Personnel

| **Personnel** | **Cost for month (USD) 2010** |
| --- | --- |
| DTS staff preparing | 714.29 |

- DTS panels preparation

| **1 Internal Quality Control** | **Cost per unit (USD) 2010** |
| --- | --- |
| Positive cryovial 1:2 | 1.12 |
| Negative cryovial | 1.00 |
| Cryovial with 700 uL buffer | 0.23 |
| Transfer pipette | 0.10 |
| Disposables gloves | 0.11 |
| Cassette of RST | 0.85 |
| Ziplock bag | 0.03 |
| Self-adhesive paper | 0.08 |
| Scissors | 0.26 |
| Photocopy | 0.04 |
| **2 External Quality Control** |  |
| Positive cryovial 1:2 | 1.12 |
| Positive cryovial 1:8 | 1.12 |
| Positive cryovial 1:32 | 1.12 |
| Negative cryovial | 1.00 |
| Cryovial with 700 uL buffer | 0.23 |
| Transfer pipette | 0.10 |
| Disposables gloves | 0.11 |
| Cassette of RST | 0.85 |
| Ziplock bag | 0.03 |
| Self-adhesive paper | 0.08 |
| Scissors | 0.26 |
| Photocopy | 0.04 |

- DTS transport

| **Transport** | **Cost (USD) 2010** |
| --- | --- |
| DTS transport | 17.86 |

- DTS staff to reconstitute

| **Staff** | **Cost for month (USD) 2010** |
| --- | --- |
| Health facility staff | 642.86 |
